# Surgical stress and cancer progression: the twisted tango

**DOI:** 10.1186/s12943-019-1058-3

**Published:** 2019-09-02

**Authors:** Zhiwei Chen, Peidong Zhang, Ya Xu, Jiahui Yan, Zixuan Liu, Wayne Bond Lau, Bonnie Lau, Ying Li, Xia Zhao, Yuquan Wei, Shengtao Zhou

**Affiliations:** 1 Department of Obstetrics and Gynecology, Key Laboratory of Birth Defects and Related Diseases of Women and Children of MOE and State Key Laboratory of Biotherapy, West China Second University Hospital, Sichuan University and Collaborative Innovation Center, 610041 Chengdu, Sichuan People’s Republic of China; 2 Deyang People’s Hospital, Deyang, Sichuan People’s Republic of China; 3 Department of Emergency Medicine, Thomas Jefferson University Hospital, Philadelphia, USA; 4 Department of Surgery, Emergency Medicine, Kaiser Santa Clara Medical Center, Affiliate of Stanford University, Stanford, USA; 5 Department of Biomedical Sciences, City University of Hong Kong, Kowloon Tong, Hong Kong, People’s Republic of China

**Keywords:** surgical stress, cancer, circulating tumor cells (CTCs), disseminated tumor cells (DTCs), ischemia/reperfusion injury (IRI), inflammation, nervous system, immunosuppression, coagulation system

## Abstract

Surgical resection is an important avenue for cancer treatment, which, in most cases, can effectively alleviate the patient symptoms. However, accumulating evidence has documented that surgical resection potentially enhances metastatic seeding of tumor cells. In this review, we revisit the literature on surgical stress, and outline the mechanisms by which surgical stress, including ischemia/reperfusion injury, activation of sympathetic nervous system, inflammation, systemically hypercoagulable state, immune suppression and effects of anesthetic agents, promotes tumor metastasis. We also propose preventive strategies or resolution of tumor metastasis caused by surgical stress.

## Introduction

**Fig. 1:**
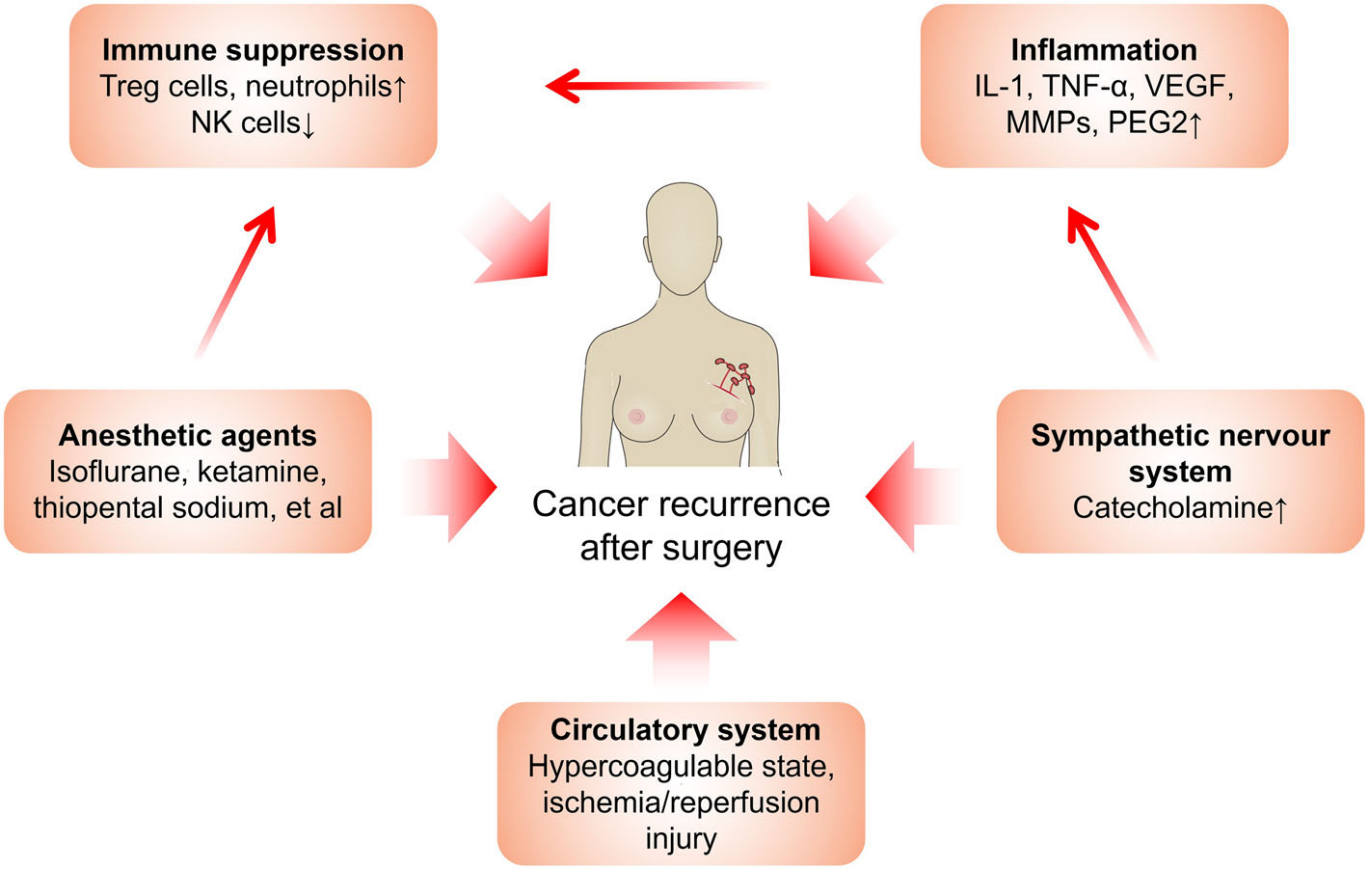
Factors that promote cancer recurrence after surgery and the interactions between them

Surgical resection remains to be a mainstay of cancer treatment. However, cancer recurs in many patients after a short time. For example, 25% to 30% of colorectal cancer patients who do not have visible evidence of metastasis during diagnosis are detected to develop metastasis within 5 years [1]. Evidence from animal and clinical trials has demonstrated that surgery-induced stress is a powerful factor promoting malignant cancer growth [2]. Surgery-induced stress is a systemic effect, involving inflammation, ischemia-reperfusion injury (IRI), sympathetic nervous system activation, and increased cytokine release, altogether significantly increasing cancer recurrence risk (Fig. 1).

**Fig. 2:**
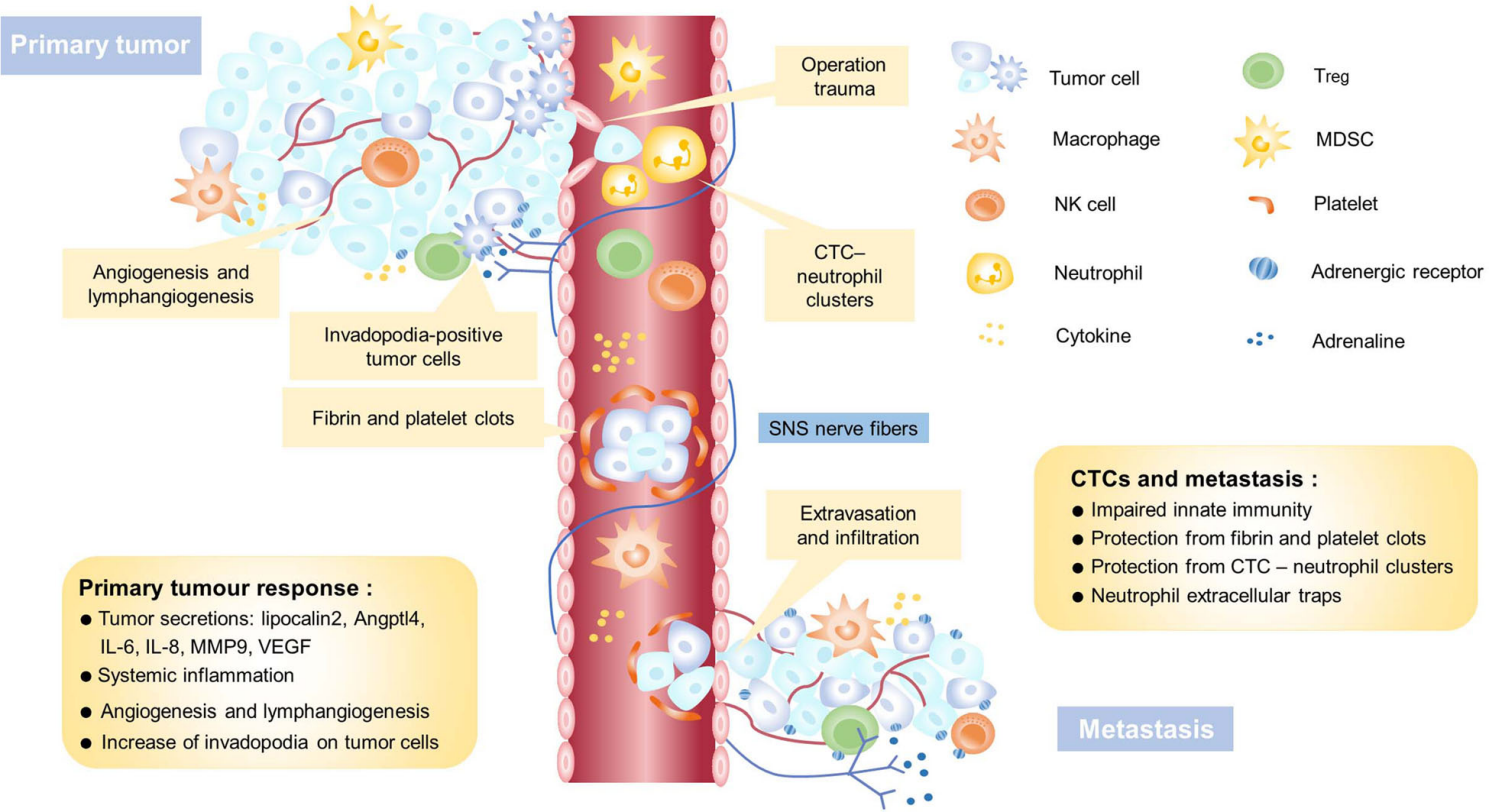
The interactions between tumor cells and the tumor microenvironment during different stages of cancer metastasis

Cells of a tumor can disseminate in peripheral blood as circulating tumor cells (CTCs), or migrate to the bone marrow or lymph nodes as disseminated tumor cells (DTCs), capable of surviving chemotherapy and initiating tumor regrowth [3]. The systemic body response to surgery may provide an environment favorable for tumor metastasis, induced by a protracted period of immunosuppression and upregulation of adhesion molecules (Fig. 2). Clinical trials have detected increased CTCs and DTCs in many cancer types, including gastric [4], lung [5], breast [6], hepatocellular [7], and colorectal [8] cancers. When tumor cells disseminate to a new environment, they may remain dormant for several years or even several decades rather than regrow immediately [9]. One recent study in breast cancer patients demonstrates such dormant cells awakening after surgery, initiating metastasis [10].

In this review, we will summarize previous research, discuss the possible effects of surgical stress on cancer progression, and analyze the responsible mechanisms. We will also present the available therapeutics that can prevent or mitigate surgical stress to improve patient outcomes.

## Surgery-induced cancer cell dissemination

Tumor cells have been known to disseminate into the vascular and lymphatic system, migrating to distant organs and initiating tumor regrowth and recurrence [3]. Clinical evidence has detected CTCs in the blood and DTCs in the bone marrow and lymph nodes; their population is an important indicator for diagnosis, prognosis, and therapeutic response in hepatocellular, breast, brain, and esophageal cancers [11–15]. CTC numbers increase following surgery for gastric [4], lung [5], breast [6], hepatocellular [7] and colorectal [8] cancers, and are associated with poor survival. DTCs found in sentinel lymph nodes have been noted to quadruple on average after breast cancer surgery [16]. Surgery promotes tumor cell migration in a complex fashion involving inflammatory factors, catecholamines, and pro-metastatic enzymes. Of note, after ‘curative’ surgery, disseminated cancer cells exhibit extreme genomic heterogeneity before initiating metastasis. This heterogeneity decreases later, and is induced by selected clonal expansion [17]. This phenomenon indicates that disseminated cells have not yet acquired all the key hallmarks of fully malignant cells.

## Ischemia/reperfusion injury (IRI)

Ischemia/reperfusion injury (IRI) often occurs in surgeries involving hepatocellular carcinoma (HCC) and liver transplantation [18, 19]. In a clinical trial of 103 liver transplant patients with HCC, IRI was associated with tumor recurrence, and extended ischemic duration exacerbated HCC recurrence risk [19]. Many signaling pathways affecting protein expression change after IRI. For instance, IRI enhances the expression of TLR9, a promoter of NF-κb and ICAM-1 [20]. The CD95/CD95L pathway, usually regarded as a pathway inducing cell death, promotes tumor cell proliferation by signaling axis such as KRAS. In the liver, the CD95/CD95L pathway is upregulated in many cell types during IRI, resulting in the apoptosis of hepatocytes and infiltrating cytotoxic lymphocytes, contributing to tumor progression [21].

IRI also modifies the secretome (e.g lipocalin2, Angptl4) of tumor cells [22, 23]. Hypoxia and inflammation could upregulate lipocalin2 (LCN2) in many cancers, promoting tumor cell survival, proliferation, and metastasis by inducing EMT (epithelial-mesenchymal transition) and eliminating the iron ion [22, 24]. In addition, IRI affects the tumor microenvironment, cultivating a better “soil” for tumor growth, migration, and adhesion. The chemokine CXCL10 secreted by monocytes, endothelial cells, and fibroblasts has a lethal effect upon tumor cells [25]. However, the endothelial progenitor cell (EPC) is mobilized by CXCL10/CXCR3 signaling after small liver grafts, promoting angiogenesis and tumor growth [26]. Matrix metallopeptidase 9 (MMP-9) is unregulated after IRI and promotes micrometastasis of colorectal carcinoma, and may represent a therapeutic target against IRI -induced tumor growth and metastasis [27, 28]. IRI also increases E-selectin, present in the endothelium, which is critical for tumor cell adhesion, and has been shown to mediate liver metastasis of pancreatic cancer [29]. Additionally, IRI contributes to tumor metastasis by its effects upon neutrophils, detailed later in this review [30].

## Sympathetic nervous system activation

The autonomic nervous system primarily regulates the body’s unconscious physiologic functions. The sympathetic nervous system stimulates the body’s fight-or-flight response, modifying blood flow and cytokine secretion [31, 32]. Sympathetic nervous system activation remains one of the most overt pathophysiological responses to surgical stress, in turn sharply increasing circulating catecholamines [33]. Tissue trauma during surgery, hypothermia, patient anxiety, metabolic derangements, and fasting all may be perioperative triggers [34].

Increase of circulating catecholamine (including adrenaline and noradrenaline) levels activates β-adrenoceptors (βAR). Activation of βAR directly affects tumor cells and indirectly remodels the tumor microenvironment [32, 35–37]. βAR activation increases metastasis in breast, lung, and colon cancer models, and accelerates growth in mammary tumors [38]. In addition, βAR activation also structurally changes tumor cells. Initial tumor cells have a defined shape and deformability. βAR activation increases the frequency of invadopodia-positive tumor cells and the number of invadopodia per cell [39]. Invadopodia are specialized actin-rich structures that facilitate invasion through the basement membrane and surrounding stroma [40]. The formation of invadopodia increases tumor cell invasiveness through the three-dimensional extracellular matrix, enhancing development of metastasis and cancer recurrence. In mammary tumors, βAR activation is associated with accelerated tumor growth [41]. In a colon carcinoma cell line, catecholamines induce *in vitro* locomotion in a β2-adrenoceptor-dependent fashion [42].

The communication between tumor cells and the microenvironment drives tumor progression [43]. Production of several pro-metastatic factors is increased in the tumor microenvironment, including matrix metalloproteinase 9 (MMP9), vascular endothelial growth factor (VEGF), IL-8, and IL-6 [44, 45]. These cytokines, increased by the autonomic nervous system activation, stimulate tumor growth by triggering inflammatory responses and promoting angiogenesis [46]. After βAR activation, inflammation-dependent mechanisms remodel tumor-associated lymphatic and blood vasculature, which in turn promote *in vivo* tumor cell dissemination [36]. Also, accumulating evidence has suggested that catecholamines have a stronger effect upon the immune system than glucocorticoids, and activation of the sympathetic nervous system suppresses natural killer cell response to tumor cells [47, 48].

## Inflammation

Surgery induces inflammation by many means, including direct wound formation [49] and infection, resulting in the release of many inflammatory mediators and the recruitment of numerous immune cell types, particularly monocytes and neutrophils [50]. Factors such as IL-1, TNF-α, VEGF, and matrix metalloproteinases (MMPs), secreted by recruited macrophages and neutrophils, all contribute to tumor progression [50, 51]. Produced by cyclooxygenase, prostaglandin E2 (PGE2) modulates various physiologic and pathologic activities, such as cell proliferation and angiogenesis [52, 53]. PGE2 promotes neoplastic progression in various malignancies. In lung cancer, PGE2 promotes metastasis by increasing MMP9 mRNA expression and inhibiting E-cadherin mRNA expression [54]. PGE2 also induces an immunosuppressive response, including increasing cancer-promoting regulatory T (Treg) cells, reducing the activated CD8^+^ T cell population, and altering the cytokines secreted by T helper cells [55, 56]. In breast cancer, PGE2 plays a key role in the “dormant-to-proliferative” transition when tumor cells disseminate to the bone microenvironment [57]. This may explain the clinical observations that inhibiting cyclooxygenase-2 (COX-2) mediates antineoplastic effects in some prostate or lung cancer patients [58, 59].

While the complement system was once regarded as an effective anti-cancer defense, significant work in recent years has identified that complement elements may promote tumor growth during chronic inflammation [60] via multiple mechanisms, such as enhancing the stemness of cancer stem cells [61], promoting angiogenesis [62, 63], and reducing anti-tumor immunity [64–66]. Surgery-induced inflammation activates the complement system, contributing to tumor recurrence. Increased levels of C3a and terminal complement complex (TCC) are observed on the second postoperative day of thoracoabdominal esophagectomy [67], a phenomenon exhibited in patients subjected to major abdominal surgery [68]. Tibial fracture surgery performed in a mouse model increases C3 levels and C3a receptor expression in hippocampal astrocytes and microglia postoperation [69]. Therefore, targeting the complement system may be an effective strategy mitigating surgical stress for cancer patients.

## Hypercoagulable state

In the normal state, few circulating tumor cells successfully colonize in new sites due to the lack of extracellular matrix support, and damage by shear stress or the immune surveillance [70]. Blood of a hypercoagulable state protects tumor cells from the above risks [71–73]. Surgery induces the release of pro-inflammatory cytokines such as IL-1, IL-6, and TNF, which increase the production of fibrinogen [71]. Besides, surgery promotes fibrin and platelet clots around tumor cells, which act as a coat of protection against detection and attack by NK cells [72, 74] and mediate tumor cell adherence to endothelial cells, releasing proangiogenic and mitogenic factors [74]. Trials demonstrate that anticoagulants can damage the fibrin/platelet coat, reduce tumor metastasis, and may significantly decrease metastatic disease after cancer surgery [72].

Platelets are crucial for hemostasis and wound healing. However, platelets contribute to tumor metastasis [75] and may be associated with decreased survival [76, 77]. A recent study demonstrates that platelets are increased in the perioperative period, and is associated with poor cancer outcomes [78]. Many mechanisms exist by which platelets are protective of tumor cells, such as cloaking tumor cells to avoid NK cell detection, promoting the arrest of tumor cells to endothelial cells, enhancing the development of secondary lesions and mediating angiogenesis [75, 79, 80].

## Immune suppressive state

Tumor cells can be protected from attack by establishing immune suppression, a long considered critical step in both tumor formation and progression [81]. Surgery provides numerous factors (inflammation, blood transfusion, and anesthetic agents) further buttressing a systemic immunosuppressive state. The immunosuppressive state after surgery can span for about two weeks [82, 83], peaking day 3 postoperative [2]. In this section, we will revisit the immunosuppressive effects of surgery, by analysis of the four main involved immune cell types.

### Regulatory T cells

Evidence supports a role for Treg cells in the establishment of immunosuppression within the primary tumor, as well as tumor cell dissemination and metastasis. Increased Treg cell population has been detected in multiple cancers, correlated with poor prognosis [84–87]. It was reported that Treg cells are markedly increased postoperatively, accompanied by decreased T helper cells and cytotoxic T cells. These factors benefit survival of neoplastic cells to varying degrees, depending upon the operative procedures performed [82, 87–89]. By unclear mechanisms, modifying T cell populations may therefore prevent cancer recurrence [90]. Hypersecretion of cortisol and overproduction of immunosuppressive acidic proteins are observed systemically after surgery [82], and may be responsible for the differential modification of T cell subpopulations.

### Myeloid-derived suppressor cells (MDSCs)

Accumulating evidence has demonstrated that number of myeloid-derived suppressor cells (MDSCs) after surgery correlates with cancer recurrence and indicates a poor prognosis [91–93]. Particularly, CD11b^+^CD33^+^HLA-DR^-^ MDSCs significantly increase in lung cancer patients after thoracotomy, and are more efficient in secreting MMP-9, promoting angiogenesis and tumor growth than MDSCs isolated before surgical operation in allograft tumor model [94]. MDSCs regulate tumor progression through various ways, including participating in the formation of premetastatic niches, promoting angiogenesis and tumor cell invasion [95]. Phosphodiesterase-5 inhibitors, such as sildenafil, could hamper the functions of surgery-derived MDSCs through downregulating the expression of arginase 1 (ARG1), IL4Ra and reactive oxygen species (ROS), enabling NK cell tumoricidal activity and reducing postoperative disease recurrence [96].

### NK cells

Surgery alters the function of NK cells, cytotoxic lymphocytes that scavenge malignant cells. In the perioperative period, natural killer cell cytotoxicity (NKC) and NK cell IFNγ secretion are both profoundly suppressed [97, 98], which might be directly induced by catecholamine, glucocorticoid (CORT), and prostaglandin (PG) [99, 100]. Surgical stress affects immune cytotoxicity by directly exerting “toxic” effects upon NK cells, which impair programmed tumor lysis in sarcoma and solid tumor patients [101]. As mentioned above, surgery also promotes the cloaking fibrin/platelet coat around tumor cells, impairing NK cell-mediated tumor clearance [72].

### Neutrophils

Protecting the body from foreign pathogens, neutrophils neutralize harmful microorganisms, and are also the main mediator of inflammation. However, neutrophils have been demonstrated to promote tumor cell metastasis both *in vitro* and *in vivo* [102, 103]. Neutrophils escort “hitchhiker” CTCs to the whole body by multiple mechanisms [3]. They can interact with CTCs and form the circulating tumor cell-white blood cell (CTC–WBC) cluster, driving cell cycle progression systemically, expanding the CTC metastatic potential [104]. Surgery induces systemic inflammation, activating neutrophils, thereby providing favorable migration conditions for CTC [105].

During microorganism invasion, neutrophils can release neutrophils extracellular traps (NETs) into the extracellular space for invader capture [106]. NETs play a positive role in innate immunity, clearing bacteria and fungi. However, NETs can trigger HMGB1 (High mobility group box 1) release, activating TLR9-dependent pathways in cancer cells, thereby promoting tumor cell adhesion, proliferation, migration, and invasion after surgical stress [107]. Metastatic dormancy has long complicated breast cancer treatment. NETs awaken dormant cancer cells in mice during inflammation, and promote the development after surgery, in the setting of increased neutrophils. As inflammation is a significant character of surgery and IRI, NETs may be implicated in surgical stress induced cancer cell dormancy revival [10, 108, 109]. It should be noted that not all effects upon the immune system are caused by surgery-induced trauma. In our next section, we discuss the anesthetic techniques reported to promote tumor growth and metastasis by reducing NK cell activity and abundance [110].

## Anesthetic agent effects

The effects of anesthetic agents upon tumor cells have been documented since the early 1980s [111]. Most anesthetic (inhaled or intravenous alike) agents contribute to tumor recurrence by directly impacting tumor cell signaling pathways or by indirectly impacting neuroendocrine and immune function [112].

### Inhalational anesthetics

Some inhalational anesthetics accelerate tumor progression. Isoflurane is a classic inhaled halogenated hydrocarbon anesthetic used for general anesthesia that inhibits activated potassium channel conduction [113]. Renal cell carcinoma cells (RCC4) proliferate and migrate more rapidly when exposed to 2% isoflurane *in vitro* [114]. Furthermore, isoflurane induces increased expression of hypoxia-inducing factors 1 and 2 (HIF1, HIF2), sequentially promoting tumor recurrence by stimulating cellular protection or primary pathogenesis of residual cells [112]. Th1:Th2 ratio [115] and NK cell activity [116] are both altered after isoflurane exposure, resulting in increased tumor cell migration [117].

Nitrous oxide acts upon a wide range of receptors [118, 119], and is associated with accelerated progression of lung and liver metastasis in mouse models. It has the strongest liver metastatic stimulation of any anesthetic studied [110, 120]. Such effects of volatile anesthesia has been recognized in clinical studies, which document markedly reduced overall survival of patients subjected to cancer surgery, even after controlling for comorbidity risk and the presence of metastatic disease [121]. This suggests limiting use of inhalational anesthetics in the setting of cancer surgery.

### Intravenous anesthetics

Some intravenous anesthetics have been demonstrated to contribute to tumor growth and metastasis, albeit *via* unclear mechanisms. Ketamine is an intravenous anesthetic which induces a trance-like state while providing pain relief, sedation, and amnesia [122] by blockade of the NMDA receptor, an ionotropic glutamate receptor [123]. Ketamine reduces the activity of NK cells, and more than doubles the survival and metastasis rate of lung tumors [124]. Increased lung tumor retention was reported after exposure to a volatile anesthetic (halothane) or intravenous agents (ketamine and thiopental) before intravenous inoculation with MADB106 breast cancer cells in rats [112].

Thiopental sodium is a highly oleophilic intravenous barbiturate anesthetic acting upon the GABA_A_ receptor channel [125]. Thiopental significantly reduced NK cell activity and increased survival of MADB106 lung tumor cells or the probability of lung metastasis [124]. Thiopental sodium inhibits the cascade reaction of NF-κB activation signal by modifying IκB kinase activity, in which the thio-group at the position of barbiturate molecule C2 plays a key role [126].

Propofol, a short-acting intravenous anesthetic of alkyl acids, has anti-cancer effects. Propofol inhibits the capacity of cancer cells for migration and invasion by impairing translation of mRNA and modulating the GTPase RhoA [127, 128]. The conjugation of propofol - DHA or propofol - EPA can significantly inhibit the adhesion (15-30%) and migration (about 50%) of breast cancer cells, and induce apoptosis (about 40%) [129]. A retrospective study from a UK group reported a 5% improved overall survival at 5 years in 2607 patients (after propensity score matching) exposed to propofol-based intravenous anesthesia compared to volatile anesthesia. Multivariate analysis by cancer type reveals that improved survival was predominantly observed in gastrointestinal and urological cancer subtypes [121].

Additionally, the method of anesthetic administration impacts cancer recurrence. Regional anesthesia (RA) refers to local anesthetic administration blocking transmission of nociceptive stimuli during tissue injury [112]. RA reduces the recurrence rate of breast cancer, prostate cancer, ovarian cancer, melanoma, and localized colon cancer, and improves overall survival rate [130]. Epidural anesthesia reduces the recurrence rate after radical prostatectomy by 57% after accounting for known prognostic factors [131].

## Therapy by pharmaceutical agents

Many pharmaceutical agents have been developed to mitigate surgical stress-induced tumor progression (Table 1). Clinical studies investigating propranolol and metoprolol demonstrate that β-blockers, which are a classical anti-hypertensive class of drugs, significantly inhibit tumor progression [132, 133]. In a clinical trial of 185 high-grade epithelial ovarian carcinoma patients, the overall survival (OS) of patients given β blockers after surgery was significantly increased compared to the control group after primary cytoreductive surgery [134]. In another trial, perioperative β-blockade significantly inhibits recurrence and metastasis of triple-negative breast cancer [135]. Patients undergoing radical mastectomy surgery exhibit increased levels of circulating epinephrine, norepinephrine, PGE 2, peripheral FOXP3 mRNA, and Treg populations; daily propranolol (60 mg) decreased Treg elevation, underlining surgery-induced catecholamines promotion of Tregs [136, 137]. Propranolol also inhibits thromboxane synthesis and reduces platelet aggregation, further contributing to its anti- metastatic properties [154]. More clinical trials assessing the effects of β-blockers upon oncologic outcomes during the perioperative period in patients with breast, ovarian, colorectal, or skin (melanoma) cancers remain ongoing [155].

**Table 1:** Therapeutic regimens to prevent cancer recurrence after surgery

Drug	Description	Perioperative anti-tumor mechanism	Examples	Reference
β-adrenoceptor antagonists (β-blockers)	Inhibitor of β-adrenoceptors; used to treat heart failure, tachycardia, and hypertension	Blockade of stress-induced catecholamine release	Propranolol, Metoprolol	[132–137]
NSAIDs	Inhibitor of cyclo-oxygenase; use to reduce pain, fever, inflammation, and prevent blood clots	Inhibition of COX-2	Aspirin, Meloxicam, Celecoxib, Parecoxib	[10, 138–141]
PDE-5 inhibitors	Inhibitor of PDE-5, conventionally used to treat erectile dysfunction	Downregulation of ARG1, IL4Ra and ROS expression	Sildenafil, Tadalafil	[96]
Immunostimulants	Many diseases such as malignancy will stimulate the immune system	Activation of immune cells (e.g. NK cells)	Toll-like receptor agonists, vaccines, checkpoint inhibitors	[142–148]
Statins	Lipid-lowering medications	Inhibition of HMG-CoA reductase or cholesterol synthesis	Fluvastatin	[149]
Anticoagulants	Inhibit thrombosis	Inhibit formation of fibrin and platelet clots	Aspirin, heparin, warfarin	[72, 150–152]
Bevacizumab	Inhibits angiogenesis	Inhibits VEGF	Bevacizumab	[153]

*Abbrevations* *: NSAIDs, nonsteroidal anti-inflammatory drugs; COX-2, Cyclooxygenase 2; NK, natural killer; HMG-CoA, β-Hydroxy β-methylglutaryl-CoA; VEGF, Vascular endothelial growth factor; PDE-5, Phosphodiesterase-5; ARG1, Arginase 1; ROS, reactive oxygen species*

Non-steroidal anti-inflammatory drugs (NSAIDs), which inhibit cyclooxygenase 1 or 2 (COX-1 or COX-2), are widely used clinically for anti-inflammatory or analgesic effect. NSAIDs effectively inhibit surgery-induced systemic inflammation, eliminating suppression of NK cell populations, preventing tumor growth and metastasis [10, 156, 157]. However, the effects of NSAIDs are very limited. For example, many reports demonstrate that celecoxib, a COX2 specific inhibitor, did not significantly affect apoptosis in prostate, breast cancer, and cervical intraepithelial neoplasia [158–161], and may only prevent colorectal adenomas [138]. Parecoxib, another COX2 inhibitor, is similarly temporally limited. Although parecoxib is an excellent analgesic in hepatocellular carcinoma [162] and has immunoprotective effect against cervical cancer [139], IL-6, IL-8, and TNF-α production is unaffected in patients receiving parecoxib 24 hours after colorectal surgery [163]. In a trial of 154 women between the ages of 25 and 65 undergoing a modified radical mastectomy for primary breast cancer, a single treatment of parecoxib did not prevent Treg elevation; propranolol plus parecoxib treatment exhibited no additive or synergistic effects compared to propranolol treatment alone [136].

Recently, rapid developments of immunotherapy have given it clinical applications. Many immunotherapy drugs inhibit surgery-induced suppression of immune cells. Toll-like receptors (TLR), which play a crucial role in activating the innate immune system, are expressed on the membranes of leukocytes and even some non-immune cells [164]. Both TLR4 agonist GLA-SE and TLR9 agonist CpG-C oligodeoxynucleotides significantly decrease cancer metastasis by increasing NK cell cytotoxicity during the perioperative period in a mouse model without adverse effects [142, 143]. Vaccines, the classic immune activators, have also been tried in combination with surgery. Perioperative treatment with influenza vaccination reversed surgery-induced dysfunction in natural killer cells and reduced postoperative metastatic disease in the mouse [144]. In another trial, oncolytic Newcastle Disease Virus (NDV) was employed to infect multiple autologous tumor cell types *ex vivo*. Postoperative injection of this OV modified tumor vaccine significantly enhanced survival in vaccinated patients compared to unvaccinated cohorts [145–147]. Checkpoint inhibitors against PD-1 mitigate postoperative T-cell dysfunction. In combination with prostaglandin inhibitors, these agents restore postoperative T-cell function completely, indicating the potential of immunotherapy in surgical stress and tumor therapy [148].

Statins, a class of agents commonly used to control hyperlipidemia, have pleiotropic effects including anti-inflammatory, antioxidative, and vasodilatatory effects, improving endothelial function, stabilizing atherosclerotic plaques, and ultimately have anti-tumor effects, albeit *via* imprecisely understood mechanisms [149]. The anti-metastatic properties of anticoagulation agents have been demonstrated in various animal models [72, 150, 151]. Antithrombotics such as aspirin, heparin, and warfarin have clinically been demonstrated to improve cancer patient survival [152], supporting their important application to prevent metastasis during surgery. Perioperative administration of bevacizumab improved survival in a clinical trial of 223 patients following lung metastasectomy for colorectal cancer [153].

## Conclusions

Surgery remains a common and important treatment for patients with solid tumors. However, despite advanced technology, new procedures, and advanced equipment availability, surgery might not significantly improve every cancer patient’s condition. In this review, we have discussed various etiologies of poor outcome in patients having undergone surgical stress during tumor removal. Innovative therapeutic solutions are in development to improve outcomes after cancer-related surgical procedures. Rigorous future evaluation of the efficacy and feasibility of these therapeutic avenues in cancer patients post operatively are warranted.

## Data Availability

Not applicable.
